# Fast Saccadic Eye Movements Contribute to the Worsened Postural Sway in Older Adults Who Have Experienced Falls

**DOI:** 10.3390/healthcare10091708

**Published:** 2022-09-06

**Authors:** Changjoon Lee, Subin Lee, Youngsook Bae

**Affiliations:** 1 Department of Physical Therapy, Shinchon Severance Rehabilitation Hospital, Seoul 03722, Korea; 2 Department of Physical Therapy, College of Health Science, Gachon University, 191 Hambangmoe-ro, Yeonsu-gu, Incheon 21936, Korea

**Keywords:** older adult, fall, postural sway, saccadic eye movement, static balance

## Abstract

The purpose of this study was to examine changes and between-group differences in postural sway during saccadic eye movement in older adults (*n* = 152). The participants were stratified into older adults who have experienced a fall (*n* = 58) (faller group) and those who have not (*n* = 94) (non-faller group). We measured postural sway during saccadic eye movement. Saccadic eye movement speed was such that the target was displayed at 0.5 Hz, 2 Hz, and 3 Hz. Postural sway was measured based on path length, velocity, and length between the maximal and minimal position of center of pressure in mediolateral and anteroposterior direction. In the faller group, path length, velocity, and mediolateral displacement of the center of pressure increased significantly during 3 Hz saccadic eye movement stimulation. However, in the non-faller group, there was no significant change in the center of pressure parameters during saccadic eye movement stimulation. Mediolateral displacement of the center of pressure increased significantly in both groups during saccadic eye movement, especially at 3 Hz. Therefore, rapid saccadic eye movement stimulation can contribute to the worsened postural sway in older adults who have experienced falls, and rapid external environmental stimuli may contribute to the deterioration of the upright standing stability in older adults.

## 1. Introduction

With advancing age, physical functions related to the ability to maintain balance start to decline, which increases the risk of falls and injury in older adults. Impaired balance in older adults is associated with multiple factors, including age-related changes in the visual, vestibular, and somatosensory systems [1]. In particular, in older adults, visual function is related to balance and the ability to perform independent activities of daily living [2].

Decreased balance ability is the leading cause of injury and death in the elderly population. Each year, 30% of adults aged >65 years and 40% of adults aged >80 years experience a fall [3]. Falls are often caused by a loss of balance ability. Performance in postural stability is usually assessed by quantifying postural sway (PS) during quiet upright standing. Quiet (static) upright standing is the assessment of balance performance and represents an individual’s ability to limit the center of pressure (CoP) within an established base of support [4]. The most commonly used PS measures are various parameters derived from the temporal pattern of the CoP, whereas traditional CoP-based measures (e.g., length and average velocity of CoP) deteriorate with age. Previous studies have reported that, in older adults, fallers have poorer postural balance than non-fallers [5,6]. Therefore, CoP may be more impaired in older adults who have experienced a fall than in those who have not.

CoP is the location point of the vertical ground reaction force vector [4]. CoP displacement plays a very important role in confirming postural control and PS [7]. Previous studies have reported decreased sway area, length, and average velocity of the center of pressure (CoP) during saccadic eye movement (SEM) both in young adults [8] and in older adults [9,10]. As such, a fast gaze-moving SEM that visually tracks a moving target from one point to another can reduce PS. This implies that the balance has improved. Therefore, SEM is an important factor used to determine the position of the body in space with respect to a moving target [11], thereby playing an important role in controlling PS in the upright posture. In a recent study, it was reported that the response of SEM to sudden external environmental stimuli may be inadequate or decreased, which may be a contributing factor to poor balance in the elderly [12]. In addition, this may be because there is a difference in balance between older adults who have experienced a fall and those who have not. Rapid SEM may decrease balance in older adults.

Despite significant research on SEM, no study has been able to identify differences in balance ability between fallers and non-fallers following SEM stimulation. In addition, SEM speed decreases with age [12], and participants may complain of dizziness when performing SEM speeds above 3 Hz [8]. Therefore, this study aimed to examine the changes in PS and differences between groups at 0.5, 2 and 3 Hz SEM stimulation. We hypothesized that 0.5, 2 and 3 Hz SEM stimuli will increase PS in the faller group but not the non-faller group and that 3 Hz SEM stimuli will worsen PS parameters.

## 2. Materials and Methods

### 2.1. Study Design

In this quasi-experimental study, data collection was conducted between June and December 2021. All study procedures were approved by the Institutional Review Board of Gachon University, and the study was conducted in accordance with the tenets of the Declaration of Helsinki. Before data collection, the patients signed a written informed consent explaining the experiment protocol. Data collection was performed at a university laboratory and community centers. All outcome measures were blinded to the purpose of this study.

### 2.2. Participants and Procedures

The participants were recruited through community center advertisements such as posters and telephonic interviews. We included adults that perform activities of daily living independently without the use of an assisted device, who had no current orthopedic problems in the lower extremities, and who were able to perform the research procedure for >30 min. Individuals who had a mini-mental state examination score of <24, did not perform SEM, whose gaze position data were not confirmed during pre-SEM training, and had previous balance impairment were excluded.

The sample size was calculated using G*Power 3.1.9 (University of Kiel, Kiel, Germany) based on a one-tailed test, power of 0.9, α-value of 0.05, and effect size of 0.5. The calculated sample size was 140. This study recruited 186 older adults. However, 14 participants did not meet the inclusion criteria, and 20 participants did not complete the research procedures due to non-confirmed gaze position or complaints of dizziness during SEM. As a result, a total of 152 older adults (aged 65–82 years) participated in this study.

**Figure 1:**
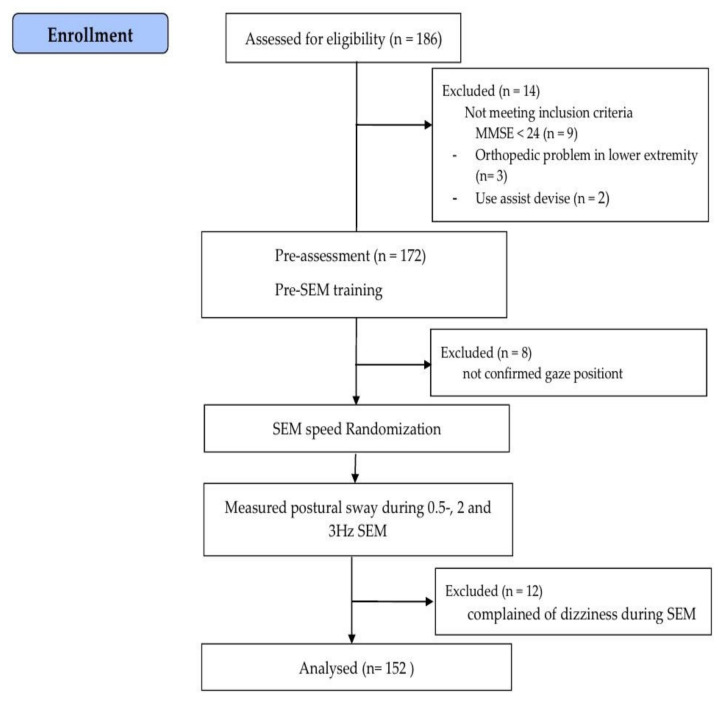
Flow chart of the study. SEM: saccadic eye movement.

The demographic characteristics were examined, including fall experience within the previous year and mini-mental state examination scores. Next, PS was measured at baseline and during 0.5, 2 and 3 Hz SEM. All participants were familiarized with how to perform SEM. The participants performed SEM randomly at 0.5, 2, and 3 Hz depending on randomization site (http://www.randomization.com, accessed on 1 January 2021) (Figure 1).

### 2.3. SEM

After pre-assessment measurements, all participants were trained on how to perform SEM (pre-SEM training). Participants sat in front of a 24-inch monitor and completed the task in 3 min. The eye movement was confirmed using an eye-tracking device (Tobii X2-30) attached to the monitor. The Tobii Eye Tracker collects 30 gaze data points per second and delivers accurate gaze position data of where the participant is staring.

The participant performed SEM by moving only the eyes without moving the body and head according to the movement of the red target displayed on the LCD monitor screen (9790 mm × 5830 mm, LG, Republic of Korea) positioned 1 m in front. The target was 2 cm, red, and was created using flash software on a white background. The baseline was measured by asking the participant to look at a target fixed in the center of the monitor so that their eyes were focused on one place. The target appeared and disappeared from one location on the screen and immediately appeared at another location. Targets were randomly displayed across the screen in diagonal, vertical, and horizontal directions. In this study, SEM rates of 0.5, 2 and 3 Hz were used, and targets appeared once per 2 s, twice per 1 s, or 3 times per 1 s at 0.5, 2 and 3 Hz, respectively [8,13].

### 2.4. Postural Sway Measurements

PS was measured in the upright posture. All participants were asked to stand barefoot on a Zebris platform (Zebris FDM 1.5, Zebris Medical GmbH; Isny im Allgäu, Germany, length × width × height, 1580 mm × 605 mm × 21 mm) in a comfortable position with arms parallel to the torso, eyes open, and feet in a neutral position. PS was assessed based on CoP displacement of the foot on the ground measured using objective instruments. The CoP parameter can detect the participant’s movement pattern and has high reliability intra-measurement [14]. CoP parameters, including path length (cm), velocity (cm/s), and mediolateral (ML) and anteroposterior displacement of CoP, were measured [8,15]. CoP data were collected and analyzed with the participants standing barefoot on a force plate. The force plate sampling frequency was set at 100 Hz.

After baseline quantification, measurements were collected according to the SEM randomization sequence. PS was measured simultaneously during 0.5, 2, and 3 Hz SEM while the participant’s gaze followed the target. In this study, SEM was performed for 50 s. The CoP was measured for 30 s from 20 s after the start of SEM. The break time between baseline and each SEM stimulation was 1 min, and participants were allowed to walk at their preferred pace [8].

### 2.5. Statistical Analysis

All statistical analyses were performed using SPSS version 26 (IBM Corp., Armonk, NY, USA). The demographic data of the participants were summarized using descriptive statistics. The general characteristics were compared between the faller and non-faller groups using the independent t-test. Intragroup differences in PS were assessed using one-way repeated measure analysis of variance (ANOVA), and the post hoc test was analyzed using Tukey’s method. For intragroup comparisons, the Bonferroni method was performed to correct for errors that may have occurred when comparing SEM trials. Based on Bonferroni correction, the new significance level was 0.05/(comparison number) and the adjusted significance level was 0.017 (with α = 0.05/3 = 0.017) [16]. For between-group comparisons, two-way repeated ANOVA was performed. The effect size was calculated as η^2^ = (Z^2/^[N-1]) to determine the significant intergroup changes. An effect size of up to 0.02, 0.13, and 0.26 indicated small, moderate, and large changes, respectively [17]. All continuous numerical variables are expressed as mean ± SD.

## 3. Results

A total of 152 people participated in this study, with faller (*n* = 58; mean age, 77.10 years) and non-faller groups (*n* = 94; mean age, 75.71 years), respectively (Table 1).

**Figure 2:**
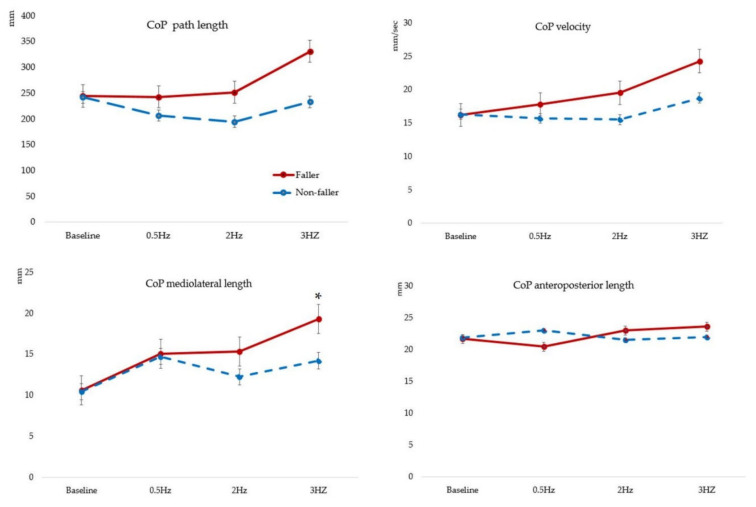
Comparisons of postural sway between baseline and during 0.5, 2 and 3 Hz saccadic eye movement. CoP; center of pressure, * *p* < 0.05 indicates a significant difference between the groups.

In the faller group, CoP_path length_ increased at 3 Hz compared with that at baseline, 0.5 Hz and 2 Hz, whereas CoP_velocity_ increased at 3 Hz compared with that at baseline. There were no significant changes in CoP_anteroposterior displacement_. CoP_ML displacement_ showed a significant increase at 0.5 Hz and 3 Hz compared with that at baseline (*p* < 0.001). In the non-faller group, CoP_ML displacement_ increased at baseline compared with that at 0.5 Hz, 2 Hz and 3 Hz. In terms of between-group comparisons, CoP_ML displacement_ decreased significantly in the faller group compared to that in the non-faller group (*p* = 0.026, η^2^ = 0.020) (Table 2, Figure 2). These results indicate that SEM stimuli at 3 Hz is associated with increased PS.

## 4. Discussion

In this study, we observed significant changes in PS based on CoP parameters following 0.5, 2 and 3 Hz SEM stimulation in older adults who were able to independently perform activities of daily living. In the faller group, CoP_pathlength_, CoP_velocity_, and CoP_ML displacement_ significantly increased. CoP_ML displacement_ also significantly increased in response to SEM stimulation. We also found significant between-group differences in this parameter.

More than 30% of adults aged ≥65 years of age experience fall each year [18], and there is an increased fall risk in older adults with poor postural balance [19]. In the present study, there was no significant difference in the CoP parameters between the faller and non-faller groups during static standing. However, the CoP_velocity_ was increased compared to the path length. Muir et al. [20] reported that the CoP displacement increased by about 21% in the elderly compared to the young, while the CoP_velocity_ increased by 82%. Therefore, the CoP _velocity_ may increase with age; we believe that the velocity is larger than the path length.

In adults of all ages, PS increases when an individual’s eyes are closed rather than open [21], suggesting that visual stimulation has a greater effect on balance. Moreover, rapid SEM can contribute to deterioration in balance ability [12]. Poor postural balance in older adults may indicate an impaired ability to recover from mild postural disturbances [22], and CoP parameters represent the response to gravity and the effects of relatively small, self-initiating corrective movements [23]. In the faller group, compared with baseline, 3 Hz SEM stimuli significantly increased CoP_path length_, CoP_velocity_, and CoP_ML displacement_. These results suggest that rapid visual stimulation can cause postural disturbances, which in turn led to a decrease in the ability to restore and maintain balance in the faller group compared with that in the non-faller group. In particular, CoP_path length_ significantly increased at 3 Hz in the faller group, but significantly decreased at 2 Hz in the non-faller group, which is similar to the results of a previous study that showed that PS decreased during 2 Hz SEM in adults [8]. Since eye movement provides information on body position, and visual signals improve the balance [24,25], the visual signals of 2Hz SEM are an effective SEM speed for reducing CoP _path length_. Additionally, CoP _ML displacement_ was reduced, but there was no difference in CoP _AP displacement_. In this study, the target displayed on the monitor mainly moved left and right, up and down, but not the front and back movement. Visual flow is a crucial signal in maintaining postural stability [25]. Therefore, we predict that only the left and right eye movements were stimulated, and the CoP _ML displacement_ was improved compared to the CoP _AP displacement_. However, there was no change in the faller group. There is a correlation between eye movement speed and balance ability, and impaired balance may also slow eye movement speed [12]. Therefore, the faller may not be able to perform eye movements that can maintain postural stability. Therefore, it was predicted that SEM stimulation can have a positive effect in reducing PS in non-fallers, although SEM stimulation increased PS in the faller group, and further research is needed to confirm the findings.

ML instability increases with age during quiet standing [26]. Increased ML sway also predicts fall recurrence [6]. Moreover, ML CoP parameters can be used to differentiate fallers from non-fallers among community-dwelling older adults [27]. Therefore, with advancing age, ML sway increases, particularly more in the faller than in the non-faller group. We found that ML sway increased during SEM stimulation for all participants compared with that at baseline. In particular, in the faller group, the CoP ML displacement increased significantly at 3 Hz SEM. In addition, the η^2^ (effect size) of the CoP ML displacement was 0.020, and the intervention effect was considered moderate when η^2^ > 0.13. Therefore, rapid SEM stimulation could worsen the CoP ML displacement in the faller. Based on these results, there is evidence that SEM stimulation increases postural instability in community-dwelling older adults who have experienced a fall. Rapid SEM stimulation further increased ML displacement and was associated with worse PS in the faller group. The elderly who have experienced falls need to be careful in real-life situations when fast saccadic eye movements occur. However, our study has several limitations. First, older adults who were able to perform activities of daily living independently were classified into faller and non-faller groups based on whether they had experienced a fall. Second, in this study, CoP parameters were measured once. The results of at least two trials should be averaged to obtain appropriate reliability for path length/average velocity. Therefore, additional research is required to investigate these specific issues. It is also necessary to confirm changes in balance ability in response to SEM stimulation in older adults at risk of falls.

Despite these limitations, this study has several strengths. To the best of our knowledge, this is the first study to confirm that fast SEM worsens PS in older adults who have experienced a fall. Therefore, SEM stimulation provides the basis for further studies to identify changes in balance ability in older adults. In addition, this study has clinical significance in that it verified balance ability through the use of different SEM frequencies.

## 5. Conclusions

Balance ability was poorer in the faller group than in the non-faller group, but there was no difference in static balance between the groups. In addition, faster eye movements deteriorated PS in the faller group. In particular, CoP_ML displacement_ increased at 3 Hz SEM in the faller group, suggesting that upright quiet balance may be worse when the eye moves rapidly in the environment.

## Figures and Tables

**Table 1:** Demographic characteristics and balance ability of the participants.

Variables	Faller (*n* = 58)	Non-Faller (*n* = 94)	t	*p*	95% CI
Age (years)	77.10 ± 6.11 ^a^	75.71 ± 5.84	1.400	0.142	1.391 (−0.573~3.354)
Height (cm)	151.76 ± 6.17	152.98 ± 16.34	−0.547	0.585	−1.224 (−5.652~3.203)
Weight (Kg)	58.75 ± 9.19	61.60 ± 10.79	−1.668	0.097	−2.846 (−6.217~0.524)
Sex (male/female)	10/48	19/75			
K-MMSE ^b^ (score)	25.91 ± 3.01	26.35 ± 2.50	−0.944	0.347	−0.441 (−1.364~0.482)
Number of falls	1.78 ± 1.09	0	14.434	<0.001	1.778 (1.534~2.022)
Postural sway of baseline					
CoP ^c^ _path length_ (mm)	244.42 ± 188.59	241.81 ± 188.35			
CoP_velocity_ (mm/s)	16.20 ± 11.71	16.32 ± 13.18			
CoP_medlateral length_ (mm)	10.56 ± 11.61	10.44 ± 9.79			
CoP_anteroposteiror length_ (mm)	21.68 ± 12.56	21.89 ± 10.71			

^a^ Mean ± standard deviation, ^b^ Korean mini-mental state examination. ^c^ CoP Center of pressure.

**Table 2:** Comparisons of postural sway between baseline and during 0.5, 2 and 3 Hz saccadic eye movement.

Variable	SEM Speed	Faller (*n* = 58)			Non-Faller (*n* = 94)			Group * Time		
		Mean ± SD	F	*p* ^a^	Mean ± SD	F	*p* ^a^	F	*p* ^b^	η^2^
CoP_path length_ (mm)	Baseline	244.42 ± 188.59	3.389	0.019	241.81 ± 188.35	0.308	0.580	2.096	0.104	0.014
	0.5 Hz	242.38 ± 177.47			206.29 ± 138.37					
	2 Hz	251.50 ± 227.14			194.64 ± 138.37 ^††^					
	3 HZ	331.03 ± 225.81 *^†‡^			232.93 ± 185.66					
CoP_velocity_ (mm/sec)	Baseline	16.20 ± 11.71	2.736	0.045	16.32 ± 13.18	0.884	0.450	0.883	0.433	0.006
	0.5 Hz	17.79 ± 12.70			15.69 ± 15.56					
	2 Hz	19.53 ± 16.49			15.51 ± 14.69					
	3 HZ	24.27 ± 27.33 ^‡^			18.73 ± 22.45					
CoP_medlateral length_ (mm)	Baseline	10.56 ± 11.61	6.821	<0.001	10.44 ± 9.79	3.387	0.019	3.108	0.026	0.020
	0.5 Hz	15.04 ± 12.53 **			14.71 ± 16.00 **					
	2 Hz	15.31 ± 13.40 ^††^			12.23 ± 11.89					
	3 HZ	19.28 ± 14.75 ^‡^			14.21 ± 12.14 *					
CoP_anteroposteiror length_ (mm)	Baseline	21.68 ± 12.56	1.514	0.213	21.89 ± 10.71	0.311	0.817	1.287	0.278	0.009
	0.5 Hz	20.46 ± 11.72			22.99 ± 15.34					
	2 Hz	23.00 ± 11.75			21.48 ± 11.10					
	3 HZ	23.59 ± 15.26			21.93 ± 15.46					

^a^ Adjusted *p*-value to 0.017, ^b^ *p*-value to 0.05, CoP; center of pressure; * *p* < 0.05 indicates a significant difference between 3 Hz and baseline; ^†^ *p* < 0.05 indicates a significant difference between 3 Hz and 0.5 Hz; ^‡^ *p* < 0.05 indicates a significant difference between 3 Hz and 2 Hz; ** *p* < 0.05 indicates a significant difference between 0.5 Hz and baseline; ^††^ *p* < 0.05 indicates a significant difference between 2 Hz and baseline. Values are expressed as mean ± standard deviation.

## Data Availability

The data associated with the paper are not publicly available but are available from the corresponding author on reasonable request.
